# Hepatitis E in Bangladesh: Insights From a National Serosurvey

**DOI:** 10.1093/infdis/jiab446

**Published:** 2021-09-22

**Authors:** Andrew S Azman, Kishor Kumar Paul, Taufiqur Rahman Bhuiyan, Aybüke Koyuncu, Henrik Salje, Firdausi Qadri, Emily S Gurley

**Affiliations:** 1 Department of Epidemiology, Johns Hopkins Bloomberg School of Public Health, Baltimore, Maryland, USA; 2 Institute of Global Health, Faculty of Medicine, University of Geneva, Geneva, Switzerland; 3 icddr,b, Dhaka, Bangladesh; 4 University of Cambridge, Cambridge, United Kingdom

**Keywords:** hepatitis E, hepatitis E virus (HEV), seroprevalence

## Abstract

**Background:**

Hepatitis E virus (HEV) genotypes 1 and 2 are a major cause of avoidable morbidity and mortality in South Asia. Despite the high risk of death among infected pregnant women, scarce incidence data has been a contributing factor to global policy recommendations against the introduction of licensed hepatitis E vaccines, one of the only effective prevention tools.

**Methods:**

We tested serum from a nationally representative serosurvey in Bangladesh for anti-HEV immunoglobulin G and estimated seroprevalence. We used Bayesian geostatistical models to generate high-resolution maps of seropositivity and examined variability in seropositivity by individual-level, household-level, and community-level risk factors using spatial logistic regression.

**Results:**

We tested serum samples from 2924 individuals from 70 communities representing all divisions of Bangladesh and estimated a national seroprevalence of 20% (95% confidence interval [CI], 17%–24%). Seropositivity increased with age and male sex (odds ratio, 2.2 male vs female; 95% CI, 1.8–2.8). Community-level seroprevalence ranged widely (0–78%) with higher seroprevalence in urban areas, including Dhaka, with a 3.0-fold (95% credible interval, 2.3–3.7) higher seroprevalence than the rest of the country.

**Conclusions:**

Hepatitis E infections are common throughout Bangladesh. Strengthening surveillance for hepatitis E, especially in urban areas, can provide additional evidence to appropriately target interventions.

Hepatitis E is estimated to cause over 3 million cases of acute jaundice each year, with more than 70 000 of these leading to death and another 3000 still births [1]. Human infections from hepatitis E viruses (HEVs), part of the orthohepevirus genus, are caused by 4 main genotypes (genotypes 1–4), with only genotypes 1 and 2 known to cause epidemics. HEV genotypes 1 and 2 are associated with self-limiting acute jaundice in the majority of infections although special populations, like pregnant women, have particularly poor outcomes with case fatality risk as high as 65% [2, 3].

While HEV was only identified in 1981 [4], retrospective analyses have identified a number of large outbreaks, which occurred on the Indian subcontinent in the 1970s and 1980s, including India and Bangladesh [5, 6]. In Bangladesh, hepatitis E is endemic with large outbreaks from time to time [7–9]. Hepatitis E is the leading cause of acute jaundice in Bangladesh and may be responsible for up to 25% of maternal mortality [7, 8, 10].

There is no effective treatment for acute hepatitis E and emergency improvements in water and sanitation have often been unsuccessful in curbing transmission [11], leaving public health workers with few effective tools to mitigate the burden of outbreaks. Fortunately, a safe and efficacious vaccine is licensed in China and Pakistan and efforts are underway for licensure in other countries and World Health Organization prequalification [12]. A phase 4 clinical trial is on-going in Bangladesh [13], but no large-scale vaccination or other HEV-specific prevention efforts are planned, in part due to our poor understanding of the burden and geographic distribution of the disease [14].

Following infection with hepatitis E, individuals develop medium- to long-lasting antibodies [15–17] that can be measured through serosurveys to provide detailed insights into the history of infection in a population. Serosurveys can help us understand the geographic distribution and magnitude of historical HEV infections, identify risk factors, and estimate key epidemiologic parameters related to transmission. Here we use a nationally representative serosurvey in Bangladesh to gain new insights into hepatitis E and provide critical details needed to target interventions, like vaccines, to areas at the highest risk.

## METHODS

### Serosurvey Design

This survey was originally conducted as part of an arbovirus study in Bangladesh with 2-stage random sampling (community and household) as previously described [18]. In brief, 70 communities from a total of 97 162 in the 2011 national census were selected with probability proportional to each community’s population. In rural areas (around three-quarters of the Bangladeshi population), the smallest administrative unit is a village, whereas in urban areas it is a ward. Within each village or ward, study staff identified the household where community leaders said the most recent wedding had taken place and selected the nearest neighbor. From this neighboring household study staff chose a random direction and counted 6 households along a transect in that direction to identify the first potential study household. For subsequent households, study staff chose a random direction and selected the sixth household from the previous household in that community. In each selected household, study staff identified the household head, described the study, and invited them to participate in the study. If the household head agreed to participate, all household members older than 6 months of age were invited to take part. Within each community, study staff visited households until the day when at least 10 households had been visited with at least 40 serum samples. Within each household study staff administered structured questionnaires with questions about household-level infrastructure, wealth, and assets in addition to individual data on demographics and travel history as well as collecting approximately 5 mL venous blood (approximately 3 mL from children aged ≤ 3 years) from all consenting individuals. Data for this survey were collected from October 2015 through January 2016.

The study was approved by the icddr,b ethics review board (protocol number PR-14058); this secondary analysis was reviewed and deemed exempt from review by the Johns Hopkins Bloomberg School of Public Health Institutional Review Board. All adult participants provided written informed consent to participate in the study. Parents or guardians of all child participants provided written informed consent on their behalf.

### Laboratory Methods

Serum samples were stored at icddr,b at −80°C before testing and then thawed to room temperature for testing. We tested 10 µL of each serum sample for the presence of anti-HEV immunoglobulin G (IgG) using the Wantai immunoassay kit (Wantai HEV IgG ELISA kit; Wantai Biological) following manufacturer’s instructions. As suggested in the packet insert, samples with a standardized optical density > 1.1 were considered positive, those < 0.9 were considered negative, and those in the range 0.9–1.1 were considered indeterminate.

### Statistical Analyses

We estimated the national seroprevalence by including survey design weights and poststratifying by age and sex to the 2011 Bangladesh census, with confidence intervals estimated using the Rao-Scott method implemented in the survey package for R [19, 20]. We used the same approach to estimating seroprevalence by urban/rural locations, sex, and age (only poststratifying by sex for age-group–specific estimates). We excluded indeterminate results from all primary analyses.

We explored the relationship between individual, household, and community-level factors and seropositivity using hierarchical logistic regression models including a spatial random field assuming a Matern covariance structure using integrated nested Laplace approximations (INLA) as implemented in the R-INLA package [21]. All individual and household-level data were collected from the survey questionnaire. Community-level data for population density [22], travel time to the nearest city [23], distance to a major water body, altitude, and poverty index [24] were collected from publicly available data sources. In the main analyses we included household and community random effects in addition to the spatial random field, but in sensitivity analyses estimated models with different combinations of random effects to understand variability in our estimates (Supplementary Table 1). We explored univariate models, a fully saturated model, a model with only variables significant in the univariate analyses, and 2 simplified models selected a priori and compared their fit with Wanatabe-Akaike information criterion [25].

Using the same INLA modeling framework we estimated seroprevalence on a 5-km by 5-km grid across Bangladesh. To do this we assigned each community to a grid cell by its centroid, estimated the mean seroprevalence in each cell containing observations, and fit spatial regression models to these data. We then used these fitted models to predict seroprevalence in the unobserved grid cells. We fit both a fully saturated model, including population size, distance from a major water body, a poverty index, travel time to the nearest city, and altitude as linear predictors in addition to spatial random effects using a Matern covariance structure and a null model with only the spatial random effects. To quantify the out-of-sample performance of this approach we used leave-one-community-out cross-validation and compared predictions to a naive model that predicted the mean grid cell seroprevalence for all but the held-out cells and calculated the mean absolute error.

We predicted seropositivity among girls reaching childbearing age (15 years) by fitting generalized additive models with penalized cubic splines to age-seroprevalence curves in each first-level administrative unit (division). We estimated simultaneous 95% confidence intervals by resampling from estimates of the variance-covariance matrix of the fitted model using a simulation-based approach with 1000 draws [26].

We used the GADM 3.6 spatial database for all administrative boundaries, which does not include boundary changes made after September 2015. All analyses were performed in R (version 4.0.2). Data and source code to reproduce analyses are available at https://github.com/HopkinsIDD/hepE-bangladesh-national-serosurvey.

## RESULTS

We tested 2924 individuals from 707 households and 70 communities representing all first-level administrative units (divisions) of Bangladesh. The median household size was 5 persons (interquartile range [IQR], 4–7), with 98% of households having more than 1 person providing a blood sample and a median of 75% (IQR, 60%–100%) of all household members providing blood. Sampled individuals had a similar age and sex distribution to the population of Bangladesh with the exception of young children, who were underrepresented [27].

Overall, 20.9% (610) of individuals tested positive for anti-HEV IgG, 78.8% (2305) were negative, and 0.3% (9) were indeterminate. After taking into account the survey design and adjusting for imbalances between the sampled population and that of Bangladesh, we estimated a national seroprevalence of 20.0% (95% confidence interval [CI], 16.5%–23.9%; design effect = 6.4).

**Figure 1.**
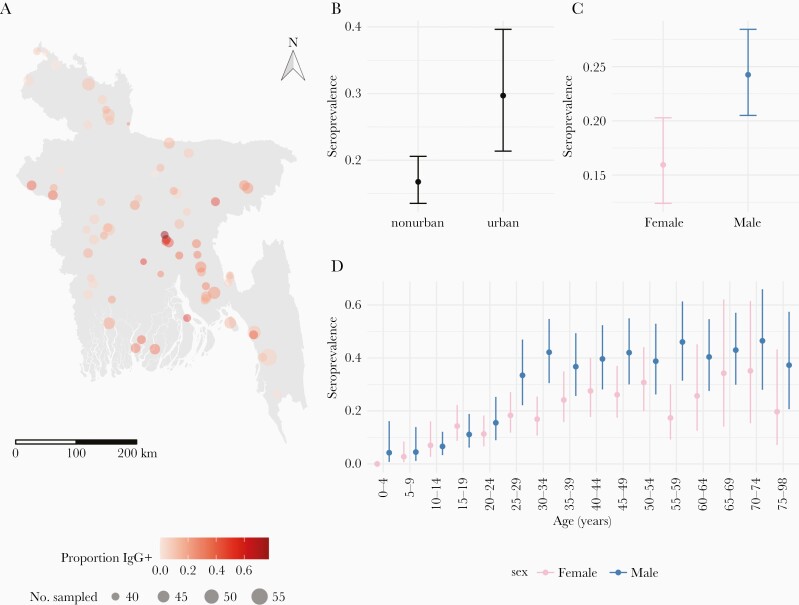
Overview of sampled individuals and communities and seroprevalence estimates. *A*, The sampled community locations, number of individuals sampled (size of dots), and the proportion of individuals seropositive (color). *B*, The adjusted seroprevalence (age/sex) by urban/nonurban classifications. *C* and *D*, The sex (adjusted for age) and age/sex-specific seroprevalence.

Seroprevalence increased with age, from 2.5% (95% CI, .6%–9.7%) in those younger than 5 years and reaching a maximum of 40.9% (95% CI, 26.0%–57.8%) among those 70–74 years old. On average, men (24.3%; 95% CI, 20.5%–28.5%) had higher seroprevalence than women (15.9%; 95% CI, 12.4%–20.3%) with this pattern holding across most age groups (Figure 1). Nationally, 90.4% (95% CI, 88.4%–92.1%) of girls reach the age of 15 years (approximately reproductive age) without evidence of antibodies and likely susceptible to disease [28].

Seroprevalence varied greatly across communities from 0% to 77.5% across the country (Figure 1). Urban areas (29.7%; 95% CI, 21.4%–39.6%) had 1.8 times higher seroprevalence than rural areas (16.8%; 95% CI, 13.5%–20.6%). Household-level seroprevalence ranged from 0% to 100% and we found no evidence of increasing household seroprevalence with household size (odds ratio = 0.99; 95% CI, .96–1.03).

While age, sex, and living in an urban area were associated with the risk of being seropositive, other community and household-level risk factors may also be important. To explore the relationship between these potential risk factors, we used a series of univariable and multivariable logistic regression models with spatially correlated errors. In univariate models (Table 1), we found significant positive associations with age, being male, travel, urbanicity, population density, and a community poverty index, and protective effects of various indicators of socioeconomic status (eg, having completed primary school compared to having no formal education, having cattle or other animals in the household, and being a household owner). However, in our primary multivariable model with spatial random effects, none of these factors were independently associated with seropositivity except for age and sex, although the effect sizes were largely consistent across various models considered (Table 1 and Supplementary Table 1). Estimates from models with varying assumptions about random effects yielded qualitatively similar results.

**Table 1.** Estimated Odds Ratios and 95% Credible Intervals for Seropositivity Including Random Effects for Household, Community, and a Spatial Random Field

Factor	Participants (n = 2896^a^)	Univariate Model, Unadjusted Odds Ratio (95% CrI)	Full Model, Adjusted Odds Ratio (95% CrI)
Individual level			
Age, y			
0–4	131 (0.05)	0.45 (.14–1.21)	0.44 (.13–1.24)
5–14	661 (0.23)	1.0 (ref)	1.0 (ref)
≥15	2104 (0.73)	6.54 (4.67–9.27)	8.73 (6.03–12.81)
Sex			
Male	1385 (0.48)	1.82 (1.50–2.22)	2.19 (1.75–2.76)
Female	1511 (0.52)	1.0 (ref)	1.0 (ref)
Travel history			
No travel in last 6 mo	1217 (0.42)	1.0 (ref)	1.0 (ref)
Travel in past wk	443 (0.15)	1.75 (1.29–2.38)	1.11 (0.76–1.60)
Travel in past mo	589 (0.20)	1.77 (1.35–2.31)	1.11 (0.81–1.53)
Travel in past 6 mo	647 (0.22)	1.45 (1.11–1.88)	1.12 (0.83–1.50)
Household level			
Household income per mo, US$^b^			
<90	308 (0.11)	0.96 (0.67–1.37)	0.91 (0.56–1.47)
91–130	531 (0.18)	1.10 (0.82–1.47)	1.12 (0.77–1.62)
131–261	1094 (0.38)	1.0 (ref)	1.0 (ref)
>261	963 (0.33)	0.97 (0.76–1.24)	0.92 (0.67–1.27)
Education, head of household			
No school	897 (0.31)	1.0 (ref)	1.0 (ref)
Primary school	744 (0.26)	0.72 (0.55–.95)	0.73 (0.51–1.04)
Secondary school	791 (0.27)	0.89 (0.69–1.16)	0.84 (0.60–1.19)
Postsecondary education	464 (0.16)	0.83 (0.61–1.13)	0.79 (0.52–1.20)
Electricity in house	2624 (0.91)	1.18 (0.78–1.81)	1.47 (0.85–2.57)
Owns land	2309 (0.80)	0.94 (0.73–1.22)	0.90 (0.63–1.27)
Owns home	2713 (0.94)	0.61 (0.40–.94)	0.66 (0.36–1.22)
Owns animals			
Pigs or rabbits	20 (0.01)	0.44 (0.11–1.44)	0.35 (0.06–1.68)
Other animals	2524 (0.87)	0.68 (0.48–.96)	0.77 (0.48–1.24)
Community level			
Urban	733 (0.25)	2.13 (1.46–3.12)	1.69 (0.88–3.24)
Distance to major water body, per 10 km	1.03 (1.58) mean (SD)	0.97 (0.86–1.11)	0.97 (0.81–1.16)
Poverty index	−0.12 (0.60) mean (SD)	1.75 (1.19–2.58)	1.35 (0.55–3.26)
Travel time to nearest city, min	12.42 (14.22) mean (SD)	0.99 (0.98–1.01)	1.01 (0.99–1.03)
Altitude, meters	16.56 (16.05) mean (SD)	0.98 (0.95–1.00)	0.98 (0.95–1.02)
Population density, log	10.72 (1.19) mean (SD)	1.25 (1.05–1.50)	1.09 (0.75–1.58)

Abbreviation: CrI, credible interval; ref, reference.

Data in the first column are No. (%) unless otherwise indicated. The full model includes all covariates shown in the table, random effects for household and community, in addition to a Matern spatial correlation function.

^a^Patients with complete data for all variables.

^b^Categories in Bangladesh Taka (TK) are < 7000, 7000–9999, 10 000–20 000, and > 20000; TK77·6 = US$1 (June 2015).

**Figure 2.**
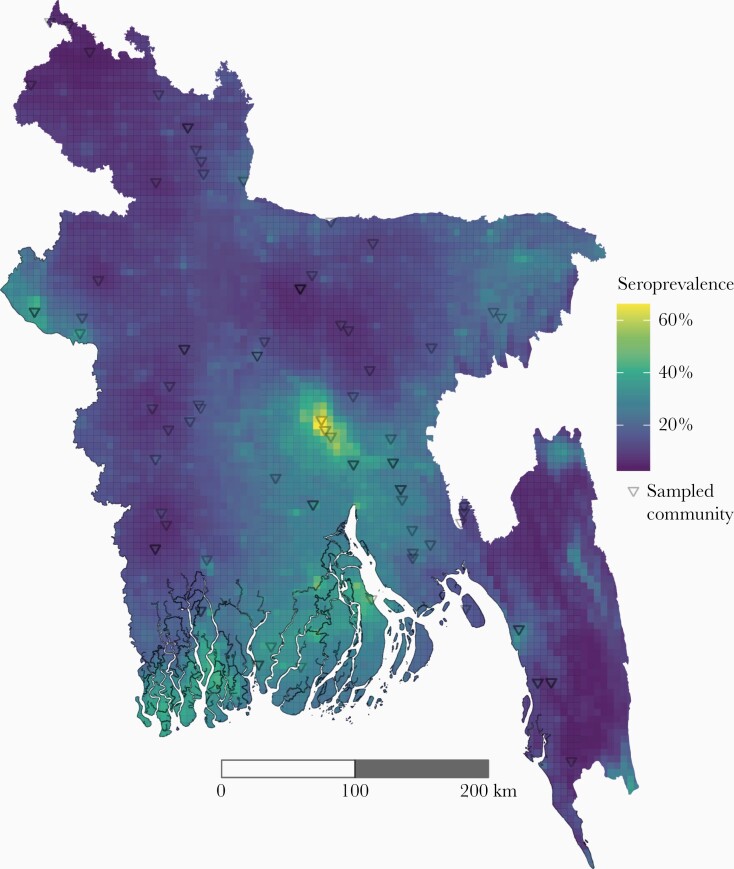
Predicted seroprevalence across Bangladesh, 2015. Predicted seroprevalence from geostatistical model with distance from major water body, population density, altitude, poverty index, and travel time to nearest city.

We fitted Bayesian geostatistical models to make a national map of seroprevalence. Our primary model demonstrated out-of-sample predictive skill (mean absolute error = 10.6%) with little bias (1.85 × 10^–4^) and moderate correlation of predictions with the true values in cross-validation (Pearson correlation) of 0.51 (Figure 2). The seroprevalence map reveals large heterogeneity in seroprevalence across the country with the highest seroprevalence around Dhaka and some evidence of higher-than-average risk in 2 other large cities, Chittagong and Rajshahi. Similar to the nonspatial analyses, we estimate from these maps that 21.6% (95% CrI, 19.0%–24.3%) of the population has been infected during their lifetime (35 177 057 people, 95% CrI, 30 919 165–39 527 957). Residents of Dhaka have a 3.0-fold (95% CrI, 2.3–3.7) higher seroprevalence than the mean seroprevalence of the rest of Bangladesh. Alternative models including different combinations of random effects (household, community, and spatial) and covariates led to similar maps.

## DISCUSSION

In this nationally representative serosurvey we found that 1 in 5 people in Bangladesh had evidence of prior HEV infection, with men having more than 1.5 times higher risk than women. Seroprevalence was 3 times higher in Dhaka, Bangladesh’s capital and largest city, than the rest of the country. Given the lack of specificity of clinical case definitions for hepatitis E (ie, acute jaundice syndrome) and limited laboratory diagnostic use across Bangladesh, our approach and results highlight an important avenue for understanding risk across the country with an aim of targeting surveillance, prevention, and control activities.

Although fecal contamination of drinking water is likely the predominant cause of HEV infections in low- and middle-income countries (LMICs) [29], measures of socioeconomic status typically associated with household access to clean water and sanitation (eg, household income, education level) were not significantly predictive of seropositivity in our study. Our data are from a study not originally designed to study hepatitis E, therefore, we did not have data on household water sources and sanitation. Despite this, our findings are consistent with existing literature on risk factors for HEV infection in Bangladesh [30, 31] and documented large outbreaks of hepatitis E associated with contaminated municipal water supplies in urban areas of Bangladesh [8]. The higher seroprevalence among men may be due to exposures outside the home, given their propensity to leave home more often than women in Bangladesh. If this hypothesis is correct, household-level water and sanitation interventions alone may not be sufficient to interrupt HEV transmission. Water and sanitation interventions may also have limited utility in preventing sporadic acute hepatitis cases associated with exposure to blood and animals, which are hypothesized to contribute to the burden of hepatitis E in Bangladesh [32] and other LMICs [33].

Samples from this same serosurvey were previously used to map the annual risk of *Vibrio cholerae* O1 infections across the country [27]. While both *V. cholerae* and hepatitis E are transmitted through fecal contamination of drinking water and food, the spatial distribution of risk of these infections are completely different in Bangladesh. For example, while many *V. cholerae* infections were estimated to occur in Dhaka, inhabitants had lower than average risk overall. In contrast, inhabitants of Dhaka had 3 times higher risk of HEV seropositivity than others in the country. Some of the differences in spatial risk profiles may be due to the fact that cholera estimates capture only a snapshot of transmission (1 year) compared to the lifetime exposures captured by HEV antibodies. Additionally, men had significantly higher HEV seroprevalence but we found no significant difference by sex for cholera. These differences might be in part explained by HEV transmission being facilitated through urban water infrastructure (eg, [8]) and cholera transmission occurring more broadly through fecal-oral routes inside households [34].

Our estimates provide a snapshot of cumulative infection risk in Bangladesh in 2015–2016. While this is useful to understand large-scale differences in risk across the country, it masks important differences in risk over time and space. The age-stratified patterns of seroprevalence, and in particular the changes in seroprevalence among the youngest children, can be particularly informative for understanding recent infection risk (ie, force of infection), which may be more important for guiding policy. Our sample size in each sampled village of children younger than 5 years was too small to permit detailed age-stratified analysis in these young age groups, although future serosurveys may benefit greatly from these individuals. Furthermore, longitudinal or repeated cross-sectional serosurveys can allow for estimates of seroincidence [31]. Estimates of the contemporary force of infection can be combined with data on the proportion of infections that become clinically apparent (and severe) to help estimate the burden of hepatitis E [1].

Given the high seroprevalence across the study population, prevention strategies such as vaccines could be valuable across the Bangladesh population. However, due to the limited supply of vaccine and costs associated with delivery, targeted vaccination strategies may be more feasible to implement than population-wide campaigns. Targeting populations at the highest risk of severe outcomes from hepatitis E infection, such as women of childbearing age who could become pregnant [8, 29], may be a cost-effective approach, especially given our results that 90% of women reach childbearing age without antibodies against the virus. While the World Health Organization suggests considering vaccine deployment in outbreaks, it has not recommended routine use of this vaccine due to limited data on the vaccine, including data on safety and efficacy of the vaccine in pregnant women and those < 16 years old [12]. Fortunately, a clinical trial evaluating the safety, immunogenicity, and effectiveness of hepatitis E vaccines among women of childbearing age, including those that go on to become pregnant, is underway in Bangladesh [13]. Data from our study suggest that these vulnerable individuals are at high exposure risk across the country, but particularly in urban areas, and the use of hepatitis E vaccines among women of childbearing age in Bangladesh may be justified.

This study comes with a number of limitations. We assumed that these serologic assays had perfect sensitivity and specificity for detecting historical HEV infections. Previous studies have estimated sensitivity and specificity of these assays to be high [33, 34]; however, without a gold standard assay to compare against these estimates are unlikely to be perfect nor generalizable to all settings. Furthermore, seropositivity has been shown to decay over time so those infected many years before the serosurvey may be differentially misclassified as seronegative [35, 36]. That seroprevalence does not significantly increase past the age of approximately 30 years old is likely due to a combination of seroreversion and changes in the force of infection over the decades. Future work synthesizing assay validation data may be valuable for correcting seroprevalence estimates appropriately. As there is only 1 HEV serotype, our estimates likely include immunologically meaningful exposures not only to genotypes 1 and 2, the most concerning for outbreaks, but other genotypes, which have not been widely described in Bangladesh and lead to different, although still severe, clinical outcomes. While we present smoothed estimates of the seroprevalence throughout the country, these are based on a geostatistical model fit to data from only 70 sampled communities throughout the country. These models assume that risk varies smoothly across space after taking into account covariates, although true HEV risk is likely less smooth over space. Finally, the household sampling approach used to recruit individuals in the parent study may have systematically excluded migrant populations and individuals living in informal settlements more likely to have inadequate water, sanitation, and hygiene (WASH) and more likely to be seropositive.

In this study we illustrate how remnant samples from population-based serologic studies not originally obtained to study hepatitis E can be an effective strategy to generate critical epidemiologic data in LMICs where surveillance infrastructure is weak or nonexistent. The severe acute respiratory syndrome coronavirus 2 (SARS-CoV-2) pandemic presents an unprecedented opportunity to leverage the increased number of representative population-based surveys to improve our understanding of the global burden of hepatitis E [38, 39]. Countries currently planning serial cross-sectional serosurveys to monitor trends in SARS-CoV-2 transmission [40] should consider utilizing remnant samples to generate data that may help quantify hepatitis E risk over time and accelerate the use of the licensed vaccine.

## Supplementary Data

Supplementary materials are available at *The Journal of Infectious Diseases* online. Consisting of data provided by the authors to benefit the reader, the posted materials are not copyedited and are the sole responsibility of the authors, so questions or comments should be addressed to the corresponding author.

jiab446_suppl_Supplementary_Table_S1

## Notes

***Acknowledgments.*** We thank all the participants of the study throughout Bangladesh for their willingness to participate in this research. We also thank the field and laboratory staff for their dedicated work.

***Disclaimer***. The funders had no role in study design, data collection and interpretation, or the decision to submit the work for publication.

***Financial support.*** This work was supported by the Centers for Disease Control and Prevention (CDC; grant number 5U01GH001207-02 original data collection). icddr,b acknowledges the commitment of the US CDC to its research efforts and thanks the Government of Bangladesh, Government of Canada, Government of Sweden, and the Government of UK for providing core or unrestricted support. National Institutes for Health (NIH; grant number R01 AI135115 ASA). F. Q. and her laboratory are supported by the NIH Fogarty International Center (grant number TW005572) and Emerging Global Leader Award (grant number K43 TW010362).

***Potential conflicts of interest*.** All authors: No reported conflicts of interest. All authors have submitted the ICMJE Form for Disclosure of Potential Conflicts of Interest. Conflicts that the editors consider relevant to the content of the manuscript have been disclosed.
